# Supplementary material for: Patterns of Intron Gain and Loss in Fungi
Source: PLoS Biol. 2004 Nov 30;2(12):e422. doi: 10.1371/journal.pbio.0020422 (PMC532390; doi:10.1371/journal.pbio.0020422)
Supplement: Table S1 — Also available at http://genes.mit.edu/NielsenEtAl/. (4.3 MB ZIP). [file pbio.0020422.st001.zip › NielsenEtAl/html/1406.html]

AN5801.1.NCU03989.1.MG04483.1.FG09865.1


```
 CLUSTAL W (1.82) Multiple Sequence Alignments - Introns Inserted


Sequence 1: NCU03989.1	338 aa
Sequence 2: MG04483.1	361 aa
Sequence 3: FG09865.1	314 aa
Sequence 4: AN5801.1	367 aa
Alignment Length: 376 aa
Number Identitical Residues: 208 aa
Alignment Score (without introns) 8997


MG04483.1 	MSS~TS-AATSSLPSPPPQSSNSTMTSHPPPPTSPGPVAAAPVV~ASIPND-TLTQ~FRG
NCU03989.1	MSA0TP-SASG-APSPLP----------------------IPTV~EKLS---AADQ~FRS
FG09865.1 	---~----------------------------------------~--------MST0FRE
AN5801.1  	MSL~NRQEATRHDGSDGVDKSALSKISQLEAP--------TSTQ0ETYPRSSLLHG~ARV
          	 :  . . ::    :   ..:  :  :   ..         .    . . .:      * 

MG04483.1 	FISQPVTAAFCAGGIAGAVSRTVVSPLERLKILFQIQSAGREEYKLSVGKALKKMWQEEG
NCU03989.1	AVSQPTVAAFCAGGVAGAVSRTVVSPLERLKILYQVQSSGREAYKLSVGKALAKMWREEG
FG09865.1 	SISQPVVAAFCAGGVAGAVSRTVVSPLERLKILMQVQSVGRDAYKLSVGKALVKMWKEEG
AN5801.1  	AVAEPVTAAFLAGGVAGAVSRTIVSPLERLKILLQIQSVGREEYKLSIWQALKKIGREEG
          	 :::*..*** ***:*******:********** *:** **: ****: :** *: :***

MG04483.1 	WRGFMRGNGTNCIRIVPYSAVQFGSYGFYKR~TLFESSPGADLTPLERLICGGIAGITSV
NCU03989.1	WRGFMAGNGTNCIRIVPYSAVQFGSYNFYKR~NIFERHPGDSLTPLSRLTCGGLAGITSV
FG09865.1 	WRGFMRGNGTNCIRIVPYSAVQFSSYNFYKR0SIFESHPGADLSPLTRLVCGGLAGITSV
AN5801.1  	WRGFLRGNGTNCIRIIPYSAVQFGSYNFYKR0-FAEPSPDADLTPIRRLICGGAAGITSV
          	****: *********:*******.**.****  : *  *. .*:*: ** *** ******

MG04483.1 	~TFTYPLDIVRTRLSIQSASFADLGDKPK--ELPGMMATMVRMYRDEGGMMALYRGIVPT
NCU03989.1	~TFTYPLDIVRTRLSIQTASFAELGERPR--KMPGMWETLVKMYRTEGGFPALYRGIVPT
FG09865.1 	~FLTYPLDIVRTRLSIQSASFAELGAKPK--KLPGMWTTLMQMYKTEGGMSALYRGIVPT
AN5801.1  	1IVTYPLDLVRTRLSIQSASFAALKRDSAGEKLPGMFTTMVLVYKNEGGFLALYRGIIPT
          	  .*****:********:**** *   . ..::***  *:: :*: ***: ******:**

MG04483.1 	VTGVAPY~VGLNFMTYEFVRTHLTPEGEKNPSAARKLLAGAISGAVAQTCTYPF2DVLRR
NCU03989.1	VAGVAPY0VGLNFMVYEHVRQYLTLDGEQNPSAVRKLLAGAISGAVAQTCTYPF2DVLRR
FG09865.1 	VAGVAPY0VGLNFMVYESVRKYLTPEGEQNPSATRKLLAGAISGAVAQTCTYPF2DVLRR
AN5801.1  	VAGVAPY0VGLNFMTYESVRKYLTPEGDSTPSALRKLLAGAISGAVAQTCTYPF2DVLRR
          	*:***** ******.** ** :** :*:..*** ******************** *****

MG04483.1 	RFQINTMSGMGYQYKSIPDAVKVIVMQEGIKGLYKGIVPNLLKVAPSMASSWLSFEVFRD
NCU03989.1	RFQINTMSGMGYQYKGIFDAVRVIVTEEGIRGLYKGIVPNLLKVAPSMASSWLSYEVCRD
FG09865.1 	RFQINTMSGMGYRYKGITDAVRVIVMQEGIKGLYKGIVPNLLKVAPSMASSWLSFEMTRD
AN5801.1  	RFQINTMSNMGYQYASIFDAVKVIVAEEGVRGLFKGIAPNLLKVAPSMASSWLSFELTRD
          	********.***:* .* ***:*** :**::**:***.****************:*: **

MG04483.1 	FFVSLDPK~ETAM----~-------
NCU03989.1	FLVGLKPE~ETKLLQ--~-------
FG09865.1 	FLVDLRPD~PEPQPL--~-------
AN5801.1  	FLLSFDER2TEVKEQYQ0CDYGHLR
          	*::.:           . .. .
```
